# The Dynamics of Neurofilament Light Chain in Spinal Muscular Atrophy

**DOI:** 10.1002/ana.78207

**Published:** 2026-03-17

**Authors:** Arlene D'Silva, Karen Herbert, Lakshmi Balaji, Jia Mei He, Tejaswi Kandula, Hugo A. Sampaio, Hooi‐Ling Teoh, Esther Tantsis, Jihee Sohn, Nancy Briggs, Nickson Ning, Matthew C. Kiernan, Didu S. Kariyawasam, Michelle A. Farrar

**Affiliations:** ^1^ Department of Neurology Sydney Children's Hospital Network Sydney New South Wales Australia; ^2^ School of Clinical Medicine University of New South Wales Medicine and Health, UNSW Sydney Sydney New South Wales Australia; ^3^ Biogen Cambridge MA; ^4^ Mark Wainwright Analytical Centre University of New South Wales Sydney New South Wales Australia; ^5^ NeuroScience Research Australia University of New South Wales Sydney New South Wales Australia

## Abstract

**Objective:**

Newborn screening (NBS) for spinal muscular atrophy (SMA) facilitates early diagnosis and treatment for affected individuals. However, fluid biomarkers that provide early insights into disease activity and outcomes in a neonatal cohort and those unable to access (due to reimbursement criteria) or deferring immediate treatment are lacking. This study evaluated neurofilament light chain (NfL) levels to provide insights into disease activity and outcomes in newborns and children with SMA.

**Methods:**

This study correlated pretreatment NfL levels in the serum and cerebrospinal fluid (CSF) in a cross‐sectional cohort of individuals with SMA against clinical, neurophysiological, molecular genetic variables, and treatment characteristics. Longitudinal NfL levels were evaluated in individuals that did not immediately commence treatment (governed by Australian reimbursement policies) and in those treated with nusinersen monotherapy.

**Results:**

Participants included 45 individuals with SMA (age range = 4 days to 42 years). Pretreatment serum NfL (sNfL) in 2 *SMN2* copy neonates were significantly higher (2 *SMN2*, mean[SE] 680.9 [163.7]; ≥ 3 *SMN2* 146.9 [59.8] pg/ml, *p* = 0.01), correlating with increasing post‐natal age (2 *SMN2 r*[12] = 0.75, *p* = 0.005). Combining sNfL and compound muscle action potential (CMAP) with pretreatment CHOP‐INTEND in a regression model provided a stronger prediction of motor outcomes for neonates at 2 years (*p* = 0.02). Pretreatment sNfL in infants with ≥3 *SMN2* copies who did not initiate immediate treatment increased despite motor function remaining stable.

**Interpretation:**

There is a malignant disease course with active denervation in children with 2 *SMN2* copies within the neonatal period. sNfL gives early insights into underlying pathophysiology prior to a clinical phenotype and may expedite access to the initiation of treatment. ANN NEUROL 2026;100:109–122

Spinal muscular atrophy (SMA) has been exemplified as a neurodegenerative condition that has been irrevocably transformed by a new diagnostic and treatment landscape.^1^, ^2^ Within this changing clinical context, there is an urgent need to identify clinically meaningful biomarkers that improve therapeutic decision making, prognostication, and treatment monitoring.

Neurofilament light chain (NfL) protein is part of the cytoskeletal architecture of motor axons, released into the interstitial fluid during axonal injury.^3^ NfL levels reflect disease activity, with significantly higher values noted in children with SMA, compared with typically developing peers.^4^ NfL has been shown to correlate with disease severity^5^, ^6^ and suppression is associated with survival motor neuron (SMN) protein repletion with disease modifying treatments. As such, it has been considered as a surrogate end point in clinical trials and for adoption into clinical care.^7^ However, before NfL is widely embedded, several aspects of its application require interrogation.^8^

This is especially pertinent as the evidence base to date for NfL in SMA has been namely accrued in children >12 months of age and with infantile onset SMA. There is a paucity of data for individuals emerging through newborn screening (NBS) programs with only 5 neonates being described internationally, including one that initiated risdiplam prenatally.^5^, ^6^, ^9^ As nearly 25% of the world's newborns are projected to have access to NBS for SMA by 2030,^10^ the specific use of NfL in a neonatal cohort requires investigation.

Real‐world data show high NfL levels in those with a 2 *SMN2* copy genotype,^11^ however, there are variable NfL levels in individuals with >2 *SMN2* copies. Acquisition of data that account for the heterogeneity of disease trajectories in children with different characteristics is vital to prognosticate long‐term outcomes and track treatment response.^8^

The nosology of SMA in a NBS era depicts a prodromal stage where presymptomatic individuals transition from clinically silent status (normal on examination) to one where subtle clinical signs and symptoms of the condition emerge (mild hypotonia and reduced tendon reflexes), a process known as phenotransition.^12^, ^13^ This phase is postulated to be underpinned by dynamic changes in the motor unit pool where relative SMN depletion may cause developmental arrest of immature motor neurons,^14^ and propagates a shift of motor neurons from reversibly dysfunctional to irreversibly damaged and degenerated states.^15^ More than half of the motor neuron pool can degenerate prior to the onset of symptoms (where there are consistent clinical features of SMA) in a process known as phenoconversion and thus the dynamic evolution of disease may be challenging to capture clinically and electrophysiologically.^16^ Here, interrogating biomarkers such as NfL that can directly link disease activity to underlying pathology, especially for those not initiating immediate treatment, are crucial to refine the current monitoring regimen^17^ and guide early treatment decision making.^18^

The primary aim of this study was to evaluate pretreatment NfL levels against disease characteristics in a cohort of individuals with SMA. The secondary aim of this study was to understand the longitudinal changes of NfL in a subgroup of individuals not accessing immediate treatment. The study also explored changes in NfL in a nusinersen‐treated population.

## Methods

### Study Design and Participants

This was a prospective cross‐sectional and longitudinal cohort study undertaken within the tertiary neuromuscular service at Sydney Children's Hospitals Network (SCHN). The study population included individuals with biallelic deletions of exon 7 on survival motor neuron 1 (*SMN1*). Participants receiving disease modifying treatments at the start of the study were excluded from enrollment, alongside those (who in the consideration of the investigator had comorbid conditions that could affect NfL levels; eg, traumatic delivery for those <28 days of life and children with clinical signs/symptoms consistent with hypoxic ischemic encephalopathy).^19^, ^20^ The prospective longitudinal arm of the study included 2 subgroups. The first were individuals who did not initiate immediate treatment in the context of dynamic approval and regulatory conditions within Australia and, second, those treated with intrathecal nusinersen as monotherapy. Ethics approval was obtained from the SCHN Human Research Ethics Committee (HREC/18/SCHN/373 and HREC/2023/ETH01937).

### Recruitment

Potentially eligible participants were consecutively recruited through the SCHN neuromuscular service from June 20, 2018, to October 30, 2024. Over the study period, the model of care changed through the commencement of NBS, enabling a heterogenous study population to be included. Those identified through prenatal or NBS and aged ≤28 days of life at enrollment formed a neonatal population. Participants older than 28 days of life were identified through NBS, clinical referral (symptomatic children), or cascade testing (family history and normal examination). All parents/caregivers or participants provided written informed consent as appropriate.

### Assessments and Outcomes

#### Biospecimen Collection and Preparation for Biobanking

All biospecimens for the study were collected during standard of care procedures for the clinical management of SMA, thus, individuals had variability in the number and frequency of samples collected (Supplementary Fig S1). Samples were stored frozen at –80°C.

## Study Measures

### Neurofilament Light Chain Analysis

NfL concentrations in cerebrospinal fluid (CSF) and serum were measured on the Quanterix Simoa HD‐X analyzer using the Simoa NF‐light immunoassay advantage kit V2 (Product 104,073, LOT number 504327 and 503,808) (Quanterix, Lexington, MA) according to the manufacturer's instructions (Supplementary Methods). Longitudinal CSF and serum samples of every participant were measured together on one plate to minimize intra‐individual variability. For quality control, 2 levels of CSF and serum endogenous control samples were prepared, and they were measured in duplicate per plate. Duplicates of the exogenous control samples were also included in the NfL kits. The mean inter‐assay coefficient of variation (CoV) was 12.1% for serum and 16.3% for CSF endogenous control pools.

### Clinical Assessments

Demographic and clinical data were collated from electronic medical records and genetic reports. This included age at NfL collection, gender, *SMN2* copy number, clinical status (presymptomatic or symptomatic), modality of diagnosis (through NBS or clinical referral), and disease‐modifying treatment details. Alongside postnatal age, postmenstrual age (PMA) was determined in the neonatal population.^21^, ^22^ Clinical status was determined through clinical examination by an experienced pediatric neurologist. Here, presymptomatic status was defined as an individual with no strongly suggestive signs or symptoms of SMA, such as hypotonia, muscle weakness, absence of deep tendon reflexes, or tongue fasciculations.

### Motor Assessments

1. A senior physiotherapist scored the Children's Hospital of Philadelphia Infant Test of Neuromuscular Disorders (CHOP‐INTEND, range = 0–64 and higher scores denote better motor function) in those <2 years old.
2. The Hammersmith Infant Neurological Examination Section 2 : Motor Milestones (HINE‐2, range = 0–26, with higher scores indicating better motor function) was completed in the newborn and infant populations, prior to treatment initiation or at the start of clinical surveillance (in untreated individuals) and after 2 years of age.^23^

### Electrophysiological Measures

Neurophysiological assessments were conducted prior to treatment and longitudinally in those being monitored (see below). Measures included motor unit potential (MUP) morphology of vastus lateralis by electromyography (EMG), and maximum compound muscle action potential (CMAP) amplitude of the abductor digiti mini muscle (ADM) after stimulation of the ulnar nerve.

### Monitoring Patients with ≥3 SMN2 Copies for Whom Treatment Was Not Initiated Immediately

When blood tests were undertaken for clinical indications, NfL was integrated with clinical monitoring of individuals who did not initiate immediate treatment. The latter was based on the recommendations of the United States SMA Expert Working Group, outlining the timing and appropriate screens and tests. These tests included motor function scales (CHOP‐INTEND, HINE‐2, Hammersmith Functional Motor Scale‐Expanded [HFMSE], and Bayley Scales of Infant and Toddler Development [BSID3]), CMAP and MUP analysis based on tolerability, age, physical ability, and clinical resources at each assessment.^23^

### Data Analysis

#### Sample Size Determination

Our sample is based on all cases seen through SCHN from June 2018 through October 2024. In this time, we recruited 45 participants. A sample of this size allows greater than 80% power to detect a difference of pretreatment NfL levels in independent group means (ie, in children with different genotypes, clinical status, and modality of diagnosis) and with an effect size of *d* = 0.86, or a significant *R* ^2^ of 0.22 in a multiple regression model with 3 predictors at alpha of 0.05. These are moderate to large effects, but effects of this size are common is this population.^24^

#### Statistics

Descriptive statistics (median, range, and proportions) were used to report sociodemographic and clinical characteristics of the cohort. Pretreatment NfL concentrations were log transformed for statistical testing based on the data distribution evaluated. The Pearson's correlation coefficient test was used to determine the relationship between CSF and serum levels. As most manufacturer immunoassay lot preparations vary by as much as ±10%, NfL concentrations generated via LOT 1 and LOT 2 NfL assays were considered interchangeable for data analysis in this study. Precision and accuracy were confirmed by carrying out precision and parallelism analyses.^25^ To overcome confounding of downstream analyses and normalize NfL data obtained from a series of experimental batches, a mean NfL value was obtained by carrying out a batch correction analyses using linear mixed models. A mixed model predicting NfL from a fixed effect of batch and a random effect for individual was specified. Mean predicted serum NfL (sNfL) value for each person was then obtained.

A strong correlation has been observed across prior studies for sNfL and CSF NfL values, determining that sNfL values can reliably replace CSF NfL measurements.^26^ For those individuals with only CSF values, imputation of serum values was carried out using linear regression. The statistical software IBM SPSS Statistics 27, GraphPad Prism 9, and R software (version 4.5.1) were used for the analyses.

Participant characteristics (including *SMN2* copy number and disease status) were used to direct biomarker analysis related to 3 clinical contexts of use; (i) before treatment initiation (in the newborn period, age ≤ 28 days and *SMN2* copy number) and infancy/childhood (age > 28 days), (ii) monitoring of participants with ≥3 *SMN2* copies for whom treatment was not initiated immediately, and (iii) treatment response with nusinersen monotherapy. Whereas age prior to treatment, *SMN2* copy number, and clinical status were accounted for in correlation analyses, sex was not considered as a confounder of NfL levels or SMA clinical presentation.^6^ Unpaired Student's *t* test was conducted to examine factors influencing sNfL levels. Results were expressed as mean ± standard error of the mean (SEM) and statistical significance was defined as *p* < 0.05 (2 tailed). Correlations between sNfL and clinical measurements were carried out using the Spearman's rank tests as scatter plots showed violations of linearity. Logistic regression and receiver operator characteristic curve analysis were used to assess sensitivity and specificity of sNfL, CMAP, and CHOP‐INTEND scores in differentiating presymptomatic and symptomatic status in the neonatal period. Published sNfL concentrations for neurologically healthy people were used to interpret sNfL levels among individuals with SMA that did not initiate immediate treatment.^19^, ^20^ As the sample size was relatively small, we used linear regression to model the relationships among sNfL, CMAP, CHOP‐INTEND, and HINE‐2 for individuals at 2 years of age. However, some models showed violations of normality or homogeneity of variance assumptions. As such, a censored normal model was used.^27^, ^28^ For analysis of the longitudinal data with nusinersen, changed NfL values were analyzed using linear models. Here, pretreatment NfL levels, age at treatment, and *SMN2* copy number were controlled for the analysis.

## Results

### Cohort Characteristics

**FIGURE 1:**
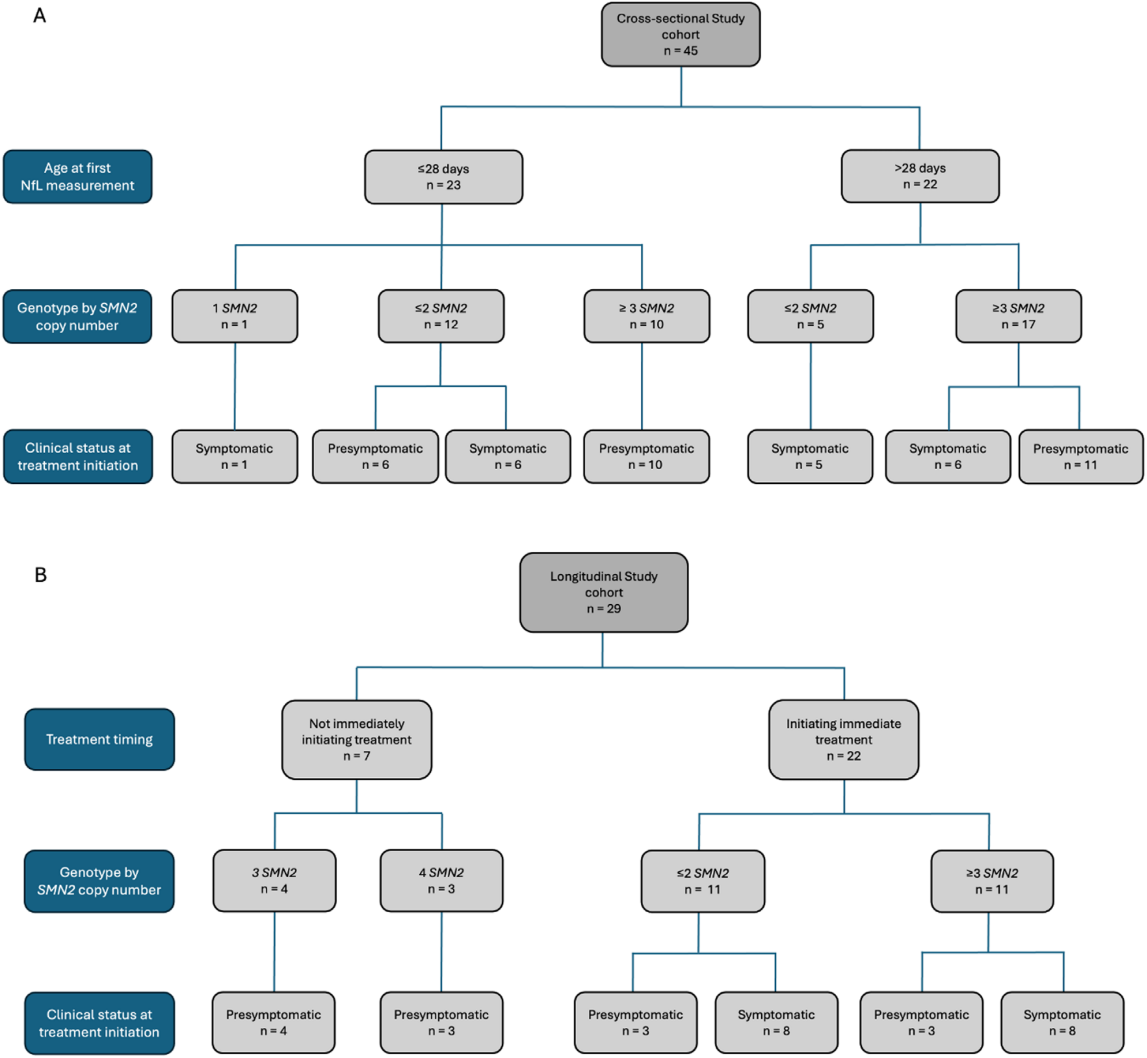
Consort diagram of individuals with SMA (A). Cross‐sectional part of the study and (B). Longitudinal part of the study. SMA = spinal muscular atrophy; *SMN2* = survival motor neuron 2. * No treatment initiated in individual with 1 *SMN2*. [Color figure can be viewed at www.annalsofneurology.org]

Of individuals meeting eligibility criteria, 95% were recruited into the study. The cohort thus included 45 individuals with SMA with a median age of 27 days (4 days to 42 years; Fig 1), with a 95% recruitment rate. The study population was heterogenous in terms of age, clinical status, disease duration, *SMN2* copy number, and motor function (Table 1). Of this cohort, 39 of 45 (86.7%) were enrolled at the time of SMA diagnosis and 6 of 45 (13.3%) had SMA symptoms prior to the availability of disease modifying therapies with a median disease duration of 8.6 years (range = 6–9.5 years). All the latter individuals had a 3 *SMN2* copy genotype.

**TABLE 1:** Demographics and Clinical Characteristics of Participants With Spinal Muscular Atrophy (SMA) at Treatment Initiation

Clinical Characteristics	Study Population (n = 45)
Sex	
M	24 (53.3%)
F	21 (46.7%)
Race	
Asian	8 (17.8%)
Black	2 (4.4%)
Middle Eastern	7 (15.6%)
White	24 (53.3%)
Other: Aboriginal	2 (4.4%)
Asian/White	1 (2.2%)
Black/White	1 (2.2%)
Modality of diagnosis	
Newborn screening	28 (62.2%)
Cascade testing, family history, normal examination	2
Clinically referred, symptomatic	5 (37.8%)
Disease status	
Symptomatic	24 (55.6%)
Presymptomatic	21 (44.4%)
*SMN2* copy number	
*1*	1 (2.2%)
*2*	17 (37.8%)
*3*	23 (51.1%)
*4*	4 (8.9%)
Age of diagnosis, days	19 (0–15,412)
Age at symptom onset, days^a^	2 (0–2,301)
Age at treatment, days	29 (0–4,075.8)
Disease duration, days	0 (0–3,468)
CMAP, mV	3.2 (0–9.05)
Age at time of study, days	27 (4–15,412)
Motor Function score (CHOP‐INTEND)	56 (7–64)

All individuals had complete data for clinical and genetic characteristics. Data are presented as number of subjects (%) or median (range). ^a^As applicable, disease duration (interval between age of symptom onset and age at first treatment). CHOP‐INTEND = Children's Hospital of Philadelphia Infant Test of Neuromuscular Disorders, reported for all participants younger than 2 years (N = 32); CMAP = compound muscle action potential (available pretreatment for 38/45 [85%], 22/23 [96%] <28 days of life); *SMN2* = survival motor neuron 2.

### sNfL and CSF NfL Levels Are Correlated in Individuals With SMA

**FIGURE 2:**
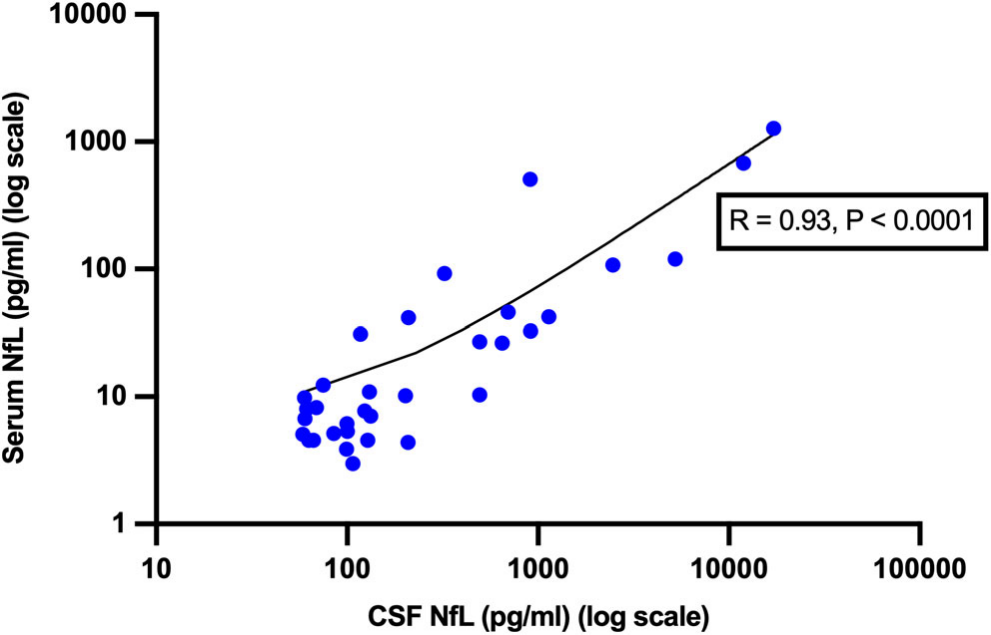
The correlation between serum and CSF NfL levels in individuals with SMA at multiple time points *r*(34) = 0.93, *p* < 0.0001. CSF = cerebrospinal fluid; NfL = neurofilament light chain; SMA = spinal muscular atrophy. [Color figure can be viewed at www.annalsofneurology.org]

**FIGURE 3:**
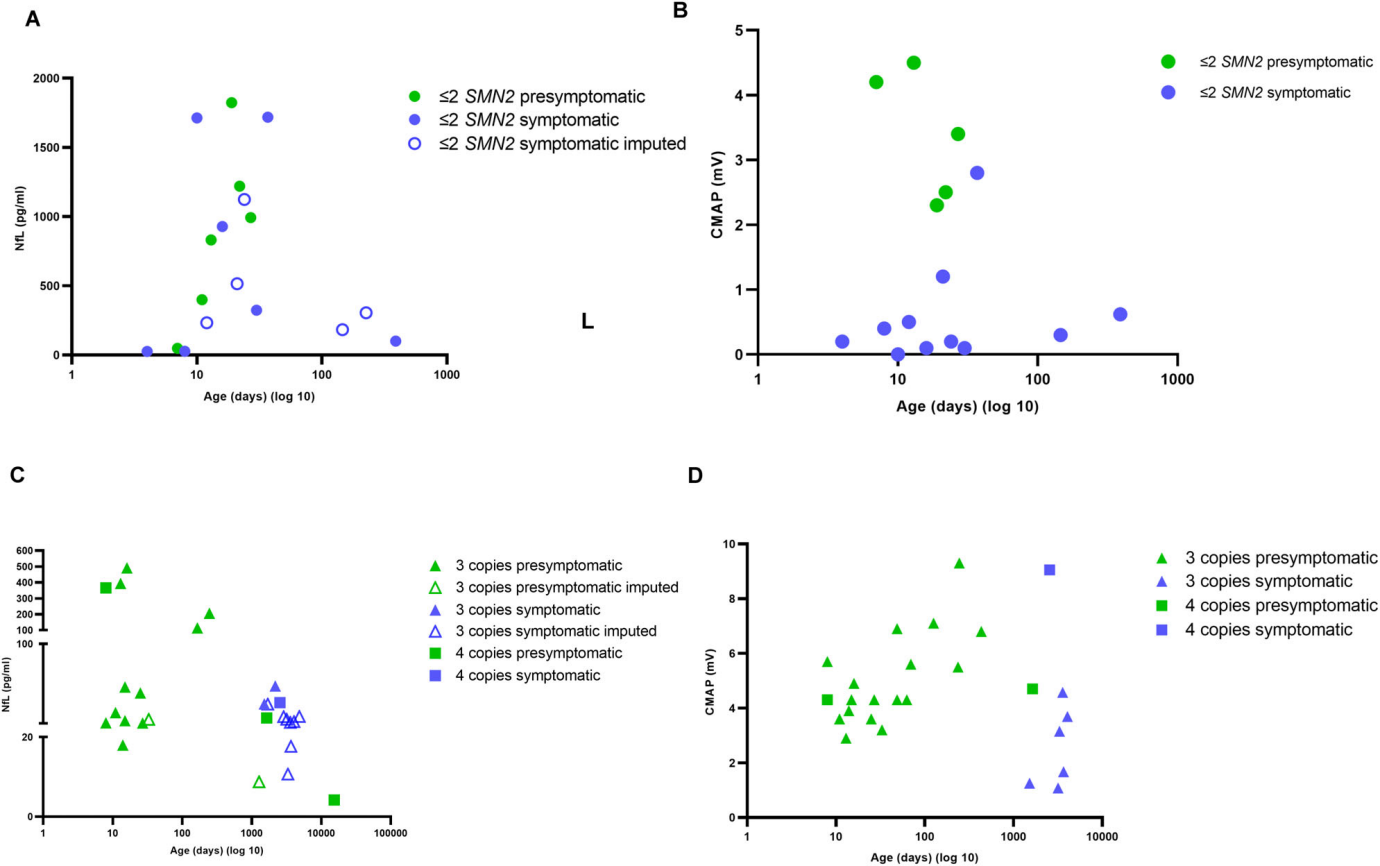
Cross‐sectional pretreatment sera NfL and CMAP measures in individuals with SMA across age according to disease status (presymptomatic = *green* and symptomatic = *purple*) (A). The sNfL levels in individuals with ≤2 *SMN2* copies and presymptomatic (n = 6), sNfL levels in individuals with ≤2 *SMN2* and symptomatic (n = 12), (B) CMAP in individuals with ≤2 *SMN2* and presymptomatic (n = 5), CMAP in individuals with ≤2 *SMN2* and symptomatic (n = 11). (C) The sNfL levels in individuals with 3 *SMN2* copies and presymptomatic (n = 13), sNfL levels in individuals with 3 *SMN2* and symptomatic (n = 10), sNfL levels in individuals with 4 *SMN2* and presymptomatic (n = 3), and sNfL levels in individuals with 4 *SMN2* and symptomatic (n = 1), (D) CMAP in individuals with 3 *SMN2* and presymptomatic (n = 18), CMAP in individuals with 3 *SMN2* and symptomatic (n = 6), CMAP in individuals with 4 *SMN2* and presymptomatic (n = 2), and CMAP in individuals with 4 *SMN2* and symptomatic (n = 1). Unfilled markers indicate imputed data points, shape and color indicate groups. CMAP = compound muscle action potential; NfL = neurofilament light chain; SMA = spinal muscular atrophy; sNfL = serum NfL. [Color figure can be viewed at www.annalsofneurology.org]

Imputation of sNfL values occurred for 15 individuals in the cross‐sectional analyses and 22 individuals in the longitudinal analyses for those treated with nusinersen. Across the cohort, CSF NfL levels were higher in comparison to sNfL levels (serum mean= 403.8 pg/ml, SE = 103.2, CSF mean = 1934.7 pg/ml, SD = 873.1, *p* = 0.02). For 20 individuals with paired samples across multiple timepoints, there was a strong correlation with sNfL and CSF NfL values *r*(34) = 0.93, *p* < 0.0001 (Fig 2). Given the high correlation observed, sNfL values for the entire data analysis were imputed from a linear regression of log serum values on log CSF NfL values. Pretreatment sNfL concentrations for the entire cohort, according to age at sNfL assessments, disease status, and *SMN2* genotypes is shown in (Fig 3).

### Pretreatment sNfL Levels in the Neonatal Period (≤28 Days)

Pretreatment sNfL levels significantly differed among 23 neonates with different genotypes (2 *SMN2*, mean[SE] 680.9 [163.7], ≥3 *SMN2* 146.9 [59.8] pg/ml, *p* = 0.01). One newborn with 1 *SMN2* copy had an sNfL of 1713.81 pg/ml on day 10 of life.

**FIGURE 4:**
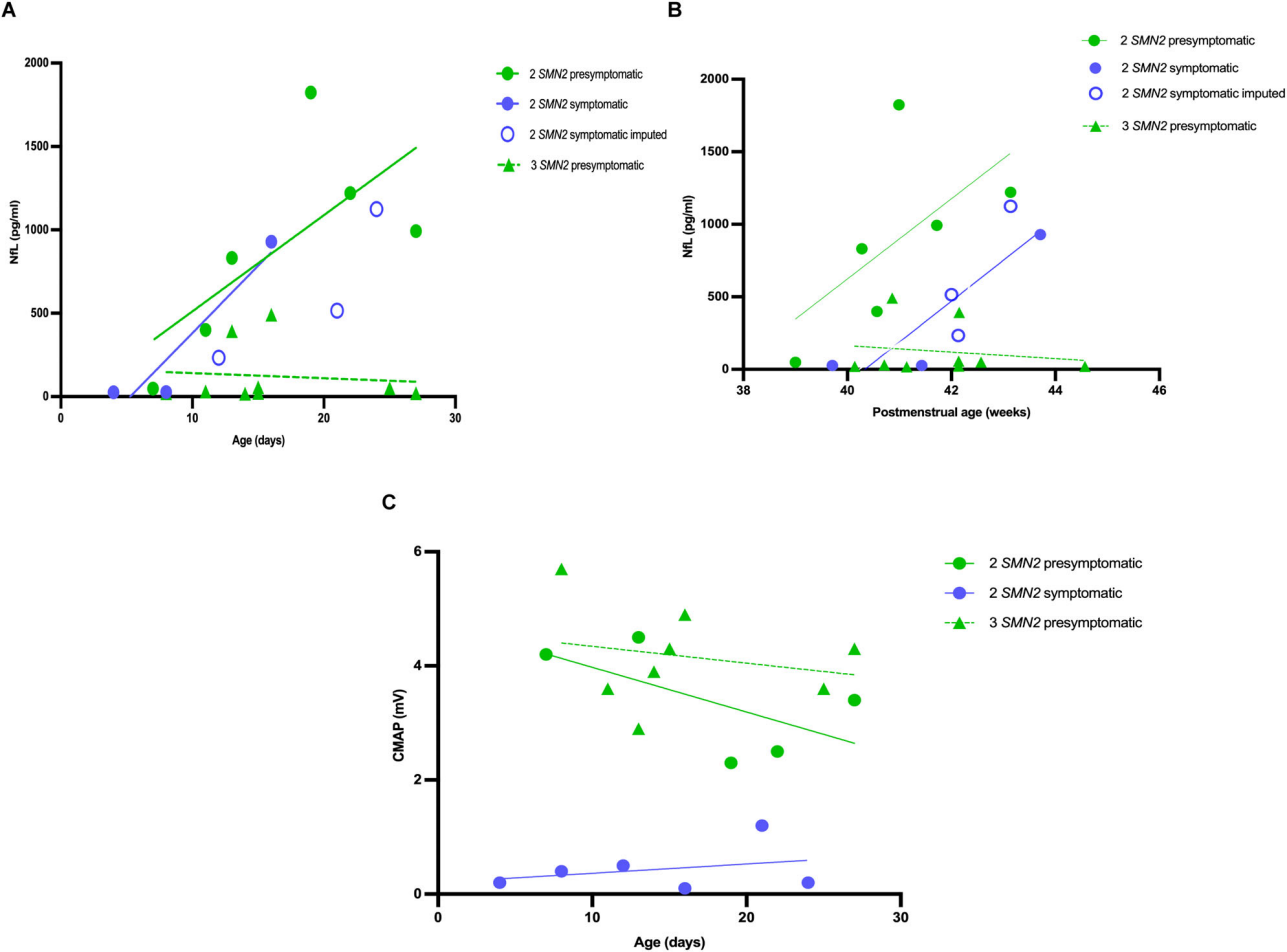
Cross sectional analysis of pretreatment sera NfL and compound muscle action potential. Cohort consists of 21 newborns (≤28 days) with 2 (n = 12) and 3 (n = 9) *SMN2* copies according to disease status (presymptomatic = *green* and symptomatic = *purple*). (A) The sNfL levels with age, 2 *SMN2* presymptomatic *r*(6) = 0.69, *p* = 0.13, 2 *SMN2* symptomatic *r*(6) = 0.86, *p* = 0.03, and 3 *SMN2* presymptomatic *r*(9) = −0.10, *p* = 0.79. (B) The sNfL with postmenstrual age, 2 *SMN2* presymptomatic *r*(6) = 0.62, *p* = 0.19, 2 *SMN2* symptomatic *r*(6) = 0.85, *p* = 0.03, and 3 *SMN2* presymptomatic *r*(9) = −0.16, *p* = 0.68. (C) Compound muscle action potential with age, 2 *SMN2* presymptomatic *r*(5) = −0.62, *p* = 0.26, 2 *SMN2* symptomatic *r*(6) = 0.31, *p* = 0.55, and 3 *SMN2* presymptomatic *r*(9) = −0.22, *p* = 0.56. Unfilled markers indicate imputed data points, shape, and color indicate groups. All participants were born at term (>37 weeks gestational age). NfL = neurofilament light chain; sNfL = serum NfL. [Color figure can be viewed at www.annalsofneurology.org]

Pretreatment sNfL concentrations significantly increased with age during the neonatal period for those with 2 *SMN2* copies (*r*(12) = 0.75, *p* = 0.005). In presymptomatic neonates with this genotype, CMAP inversely correlated with age *r*(5) = −0.62, *p* = 0.26, although not statistically significant (Fig 4).

For participants with 3 *SMN2* copies, pretreatment sNfL levels (*r*(9) = −0.10, *p* = 0.79) and CMAP amplitudes (*r*(9) = −0.23, *p* = 0.56) were not correlated with age in the neonatal period. Similar patterns for sNfL levels were observed with post menstrual age (see Fig 4). Of note, lowest sNfL levels (33.9 [7.2] pg/ml) were noted in 3 individuals predicted to have a severe phenotype (2 *SMN2*) all measured within the first 10 days of life, of whom 2 were symptomatic.

### Pretreatment sNfL Levels After the Neonatal Period in Symptomatic Infants and Children Prior to Disease‐Modifying Therapy

There were 16 of 45 (35.5%) symptomatic individuals who had sNfL levels obtained after the first 28 days of life. After the neonatal period, pretreatment sNfL concentrations were significantly different between symptomatic individuals with different genotypes (2 *SMN2* 526 [300.9], ≥3 *SMN2* 29.7 [4.0] pg/ml, *p* = 0.02) and significantly changed with age for individuals with ≥3 *SMN2* copies (2 *SMN2* copies *r*(5) = −0.6, *p* = 0.32), and ≥3 *SMN2* copies (*r*(11) = −0.6, *p* = 0.03).

Across all children diagnosed with SMA through NBS, the respective sensitivities and specificities for differentiating clinical status in the NBS cohort for sNfL were 62.5% (95% confidence interval [CI] = 25–87.5) and 80% (95% CI = 60–95), CMAP 100% (95% CI = 100–100) and 89.5% (95% CI = 73.7–100), and CHOP‐INTEND scores 87.5% (95% CI = 62.5–100) and 90% (95% CI = 75–100). A combined analyses incorporating sNfL, CHOP‐INTEND, and CMAP was carried out. For CHOP‐INTEND and sNfL, an overlap in the distributions for the 2 groups was observed. However, for CMAP, there was near‐complete separation; that is, almost all individuals with symptomatic status had a CMAP of <2 mV. Thus, CMAP itself was almost a perfect predictor of disease status.

### Monitoring to Detect Disease Activity in Presymptomatic Individuals With ≥3 SMN2 Copies for Whom Treatment Is Not Immediately Initiated

Seven individuals were presymptomatic, did not initiate treatment immediately following genetic diagnosis of SMA (governed by Australian reimbursement policies at the time), and were monitored longitudinally to detect a change in clinical status (phenotransition or phenoconversion). This included 4 individuals with 3 *SMN2* copies and 3 with 4 *SMN2* copies (Supplementary Tables S1 and S2).

The sNfL trajectories differed in presymptomatic individuals between those with 3 and 4 copy numbers (Table 2). During infancy, pretreatment sNfL levels increased among those with 3 *SMN2* copies, differing from the significant association between decreasing sNfL and increasing age observed in neurologically healthy children.^29^ Notably, over 8 weeks, sNfL levels increased 17‐fold, concurrent with subtle symptoms and a neurogenic EMG.

**TABLE 2:** Longitudinal sNfL Levels in Clinically Silent Individuals With ≥3 *SMN2* for Whom Treatment was not Initiated Immediately

Participant	*SMN2*	Clinical Symptom/Signs of SMA, Age	Electrophysiology Features During Monitoring	Treatment	Pretreatment NfL pg/ml, 95th Centile for Age, Age of NfL^25,26^	Post‐Treatment NfL pg/ml, 95th Centile for Age, Age at NfL	Age and Values at Last Clinical/Motor Assessment
1	3	Tremor and head lag at 2.5 mo	EMG neurogenic at 2.5 mo	Yes at 2.3 mo, risdiplam^a^	3,076 (18.2), 0.4 mo	8 (18.3), 4 mo	27 mo
					522 (18.2), 2.5 mo	5.1 (18.3), 6 mo	BSID 7 (1 SD below mean)
2	3	Nil	No	Yes at 8 mo, OAV^b^	205.6 (18.2), 8 mo	315.2 (18.3), 10.5 mo	32 mo
							BSID 17 (2 SD above mean)
							HFMSE 62
3	3	Nil	EMG not performed	Yes at 14 mo, OAV^b^	113.7 (18.2), 2.3 mo	84.4 (15.0), 29 mo	66 mo
					232.2 (16.6), 14 mo	195.6 (15), 53 mo	HFMSE 66
4	3	Nil	No	Yes at 42 mo, nusinersen^b^	8.8 (15), 42 mo	13.2 (15), 44 mo	84 mo
							HFMSE 66
5	4	Nil	CMAP and EMG not performed	Loss to follow up aged 66 mo	30.8 (18.6), 10 mo	NA	54 mo
					18.8 (12.4), 54 mo		
6	4	Nil	EMG neurogenic at 9 mo	Yes at 10 mo, risdiplam^a^	19 (18.6), 9 mo	12 (18.6), 11 mo	1 mo
							BSID 13 (1 SD above mean)
7	4	Nil	No	No, undertaking clinical monitoring	4.7 (10), 504 mo	NA	552 mo
					4.2 (10), 552 mo		HFMSE 66

^a^ The identification of neurogenic changes on EMG fulfilled contemporary reimbursement criteria.

^b^ Presymptomatic treatment initiated due to changing access pathways. NfL was collected concurrent with blood tests undertaken for clinical indications in line with study protocol. The 95th centiles established in neurologically healthy individuals include: ages 2–12 months =18.2 pg/ml; 12–24 months = 16.6 pg/ml; 24–48 months = 15.0 pg/ml; and 48–60 months 12.4 pg/ml^25^ and for adults = 10 pg/ml^26^ (age <2 mo not available).

BSID3 = Bayley Scales of Infant and Toddler Development 3; CMAP = compound muscle action potential; EMG = electromyography; HFMSE = Hammersmith Functional Motor Scale Expanded; NfL = neurofilament light chain; OAV = onasemnogene abeparvovec; SD = standard deviation; SMA = spinal muscular atrophy; sNfL = serum neurofilament light chain.

Two presymptomatic children with 4 *SMN2* copies had NfL levels >95th centile for neurologically healthy age‐matched controls at 10 months and 4.5 years. Three individuals initiated treatment within the presymptomatic stage due to changing access pathways, and for another 2 the identification of neurogenic changes on EMG fulfilled contemporary reimbursement criteria (one had subtle signs consistent with the prodromal phase) and started treatment; all demonstrated motor function outcomes consistent with expectations for typically developing children (follow‐up = 4.5 to 55.4 months post‐treatment initiation). For those that initiated nusinersen (n = 1) or risdiplam (n = 2), post‐treatment sNfL levels decreased over time or remained within the healthy control range, whereas sNfL increased following onasemnogene abeparvovec (n = 2).

### Evaluating the Role of Biomarkers to Predict Motor Outcomes

Univariate analyses of pretreatment variables in newborns were not associated with functional motor changes as denoted through change in HINE‐2 at 2 years of age; CMAP (*B* = −0.11, 95% CI = −0.27 to 2.45, *p* = 0.93), sNfL (*B* = −0.19, 95% CI = −3.01 to 2.62, *p* = 0.88), or CHOP‐INTEND (*B* = 0.195, 95% CI = −0.159 to 0.549, *p* = 0.25). In contrast, multiple regression analysis using a censored normal model, which included sNfL and CMAP, showed that pretreatment CHOP‐INTEND was a significant predictor of change in HINE‐2 over 2 years (*B* = 0.873, 95% CI = 0.155–1.591, *p* = 0.02). This regression did not adjust for other variables due to small sample size (n = 8).

### sNfL Levels Are Reduced in Children Treated with Nusinersen as Monotherapy

**FIGURE 5:**
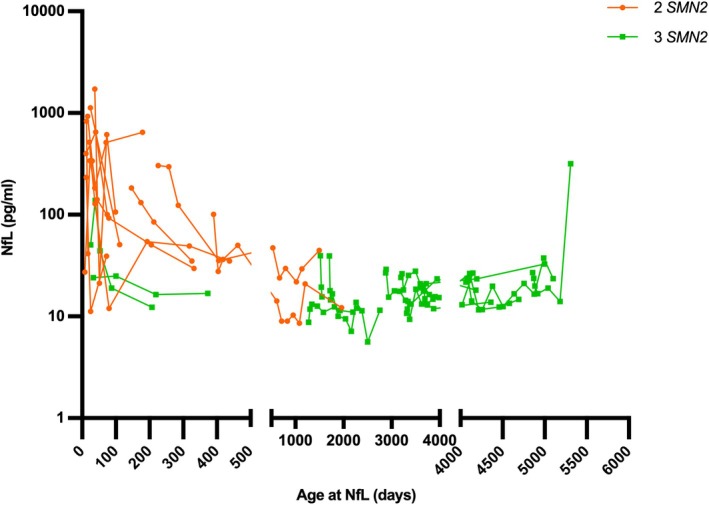
Changes in sNfL levels with nusinersen monotherapy across the cohort (n = 22) with 2 *SMN2* copies (*orange*) and 3 *SMN2* copies (*green*). All samples were taken at the time of nusinersen administration. sNfL = serum neurofilament light chain. [Color figure can be viewed at www.annalsofneurology.org]

For 22 of 45 (48.9%) of the cohort accessing nusinersen treatment, longitudinal sNfL values were evaluated. Of these individuals, 19 of 22 (86.4%) showed reduction of sNfL levels during the induction phase (first 4 loading doses) of nusinersen treatment (Fig 5). Lower levels of sNfL were noted at treatment initiation for the 3 individuals who did not show reduction in sNfL levels with nusinersen treatment (day 0– day 63). Of note, 2 of these individuals were presymptomatic at treatment initiation, whereas the sNfL level for the third individual was assessed at day 8 of life.

A greater reduction of sNfL following nusinersen was observed in those initiating treatment in the neonatal period in comparison to those in infancy/childhood (neonatal period ΔsNfL 405.3 pg/ml [178.2]; infants/children ΔsNfL 143.0 pg/ml [113.9], *p* = 0.20). The magnitude of sNfL reduction following nusinersen was significantly greater in those with 2 *SMN2* copies (2 *SMN2* ΔsNfL 468.6 pg/ml [173.8], 3 *SMN2* ΔsNfL 8.2 pg/ml [3.2], *p* = 0.01).

For individuals with a HINE at 2 years and treated with nusinersen (n = 13), greater reduction of sNfL over the induction phase was associated with greater gains in HINE‐2 at 2 years of age (*B* = 3.14, 95% CI = 0.13 to 6.15, *p* = 0·042), when controlling for pretreatment sNfL values. When including *SMN2* copy number and age of treatment, this association was not maintained (*B* = 1.83, 95% CI = −1.91 to 5.57, *p* = 0.29). The point estimate, however, was sizable, and the standardized coefficient indicated a large effect (*β* = 0.48), thus the small sample size resulted in the low power for this effect.

## Discussion

**FIGURE 6:**
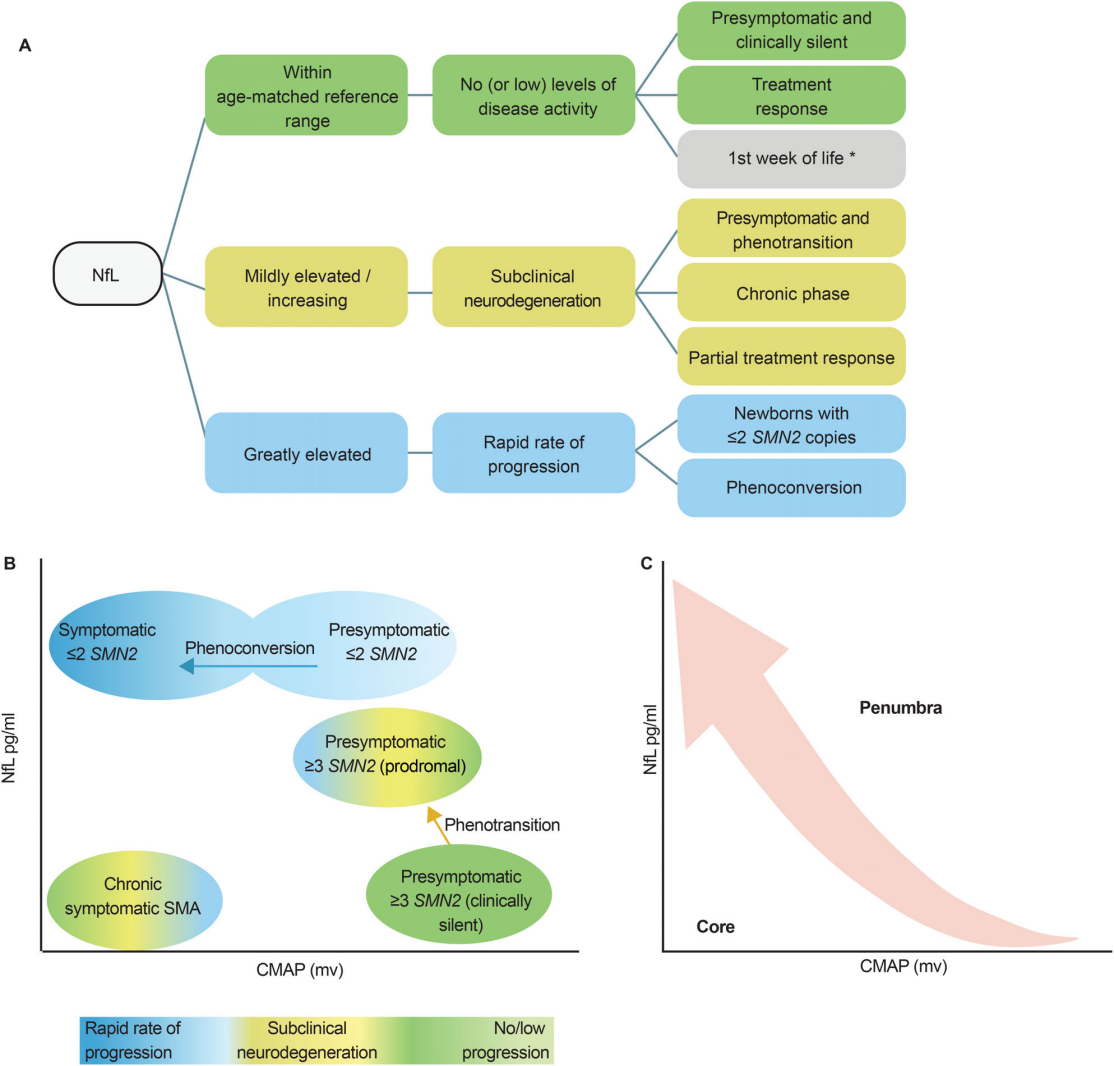
Practical interpretation of NfL levels in SMA (A). Proposed algorithm for clinical context of use. (B) Summary of pretreatment NfL levels when compared with clinical, neurophysiological, and molecular measures. (C) Conceptualizing the core‐penumbra mismatch. CMAP = compound muscle action potential; NfL = neurofilament light chain; SMA = spinal muscular atrophy; *SMN2* = survival motor neuron 2. [Color figure can be viewed at www.annalsofneurology.org]

This study fulfils international research priorities by generating real‐world knowledge on the clinical context of use of NfL.^8^, ^26^ Whereas NBS has transformed SMA,^30^ our study emphasizes biological heterogeneity, and the challenges of ascribing “presymptomatic” status based on clinical assessment alone. Our findings suggest that NfL may be useful as an early biological warning sign of disease activity and monitoring, especially in an emerging NBS population but also in children within the prodromal and chronic phases of disease. The sNfL appears to have the greatest use when adjunctive to clinical and electrophysiological measures, refining the clinical surveillance paradigm for untreated individuals^17^ by giving earlier insights and detection of different SMA disease stages including the understudied phase of phenotransition (Fig 6).

Within a presymptomatic cohort with 2 *SMN2* copies, the universally rising trend of sNfL and concurrent steep decline of CMAP within the first month of life, reflects a highly active, early onset disease process.^31^ This emphasizes the concept that, although 50% of newborns with 2 *SMN2* copies appear presymptomatic,^32^ for all children with this genotype in the neonatal period, a malignant neurodegenerative process is at play. These neonates rapidly enter a state of phenoconversion from presymptomatic to symptomatic, highlighting not only the necessity of treatment, but the urgency to intervene at the earliest possible juncture postnatally. These data thus emphasize the need for early and potential prenatal treatment in children with this genotype, to ameliorate the consequences of SMN depletion.^9^, ^33^ This knowledge may inform the rationale for bridging therapies to minimize unnecessary delays to treatment, whereas the clinical trial landscape continues to explore the role and long‐term effectiveness of combining treatment modalities. With SMA carrier screening becoming relevant in some health care jurisdictions the study generates further evidence of a highly active disease process to plan obstetric care around timely therapeutic interventions for known affected families, especially those with the 2 *SMN2* copy genotype.^9^

Our findings suggest that electrophysiological and motor function measures are more sensitive at detecting this point of disease evolution in an NBS population. Thus, current protocols for disease stratification into clinical trials based on CMAP or motor function remain highly relevant.^34^ In contrast, for most neonates with ≥3 *SMN2* copies, sNfL and CMAP levels remain stable or rise gradually within the newborn period, suggesting initial noolower levels of disease activity.

The management of presymptomatic individuals predisposed to SMA is governed by reimbursement policies, which vary globally. Monitoring to detect and treat disease activity during the prodromal phase is important for a large proportion of the global population unable to access or deferring immediate treatment. Individuals with >3 *SMN2* copies, show no or minimal changes in clinical and functional measures. CMAP levels also remain relatively stable or rise despite disease progression perhaps due to collateral reinnervation processes, that can compensate for >50% loss of the motor unit pool.^35^ In contrast, prior to treatment, high or rising sNfL levels which are >95th centile for age‐matched healthy controls may herald subclinical neurodegeneration to provide early prescient insights into the disease course which other biomarkers may not be able to capture. Here, for those presymptomatic individuals not initiating immediate treatment, a measurable indicator of underlying pathophysiology prior to the emergence of a clinical phenotype, such as sNfL, can improve monitoring, therapeutic decision making, and outcomes.

The study ascertains that there is no one size fits all and instead biomarker selection and relevance should be guided by the clinical context. As a further example, interpretation of sNfL values is driven by age, as noted by the heterogeneity of values for neonates within the first week of life in this study. Here, the role of maternal factors (renal and placental clearance) within the first 7 days of life requires further elucidation. Cumulatively, the findings in the neonatal subgroup emphasize that a precision medicine paradigm is evolving where based on the individuals’ genotype and clinical characteristics a platform of clinical, molecular, and/or electrophysiological biomarkers are available to denote disease evolution or clinical status.

For individuals with 4 *SMN2* copies, longitudinal sNfL assessments may provide a window of understanding into the timing and extent of disease progression, which have hitherto been assessed from a symptom perspective only and variably ascertained in real‐world data.^13^ Here, low levels of sNfL are observed with age, however, these remain higher than pre‐established normative ranges but lower than for children predicted to have a severe, infantile onset form of the disease. Cumulatively, this may denote a subtle process of underlying disease progression despite a clinical appearance of stability. On the other end of the spectrum, sNfL levels that remain lower than normative ranges over time are noted in older children and adults who remain clinically presymptomatic with the same genotype. This knowledge generation is highly relevant as there is a paucity of international evidence on disease course for children with 4 *SMN2* copies, precluding the ability to instigate treatment at the correct juncture. Our study affirms that sNfL, alongside CMAP and EMG studies, can reflect neurogenic changes with this genotype and support stratification of individuals into treatment or clinical surveillance pathways. Importantly, with NfL being used in clinical trials as a surrogate end point, validation in real‐world cohorts, as in our study, may more accurately target and denote the earliest timepoint of intervention, prior to clinical signs and symptoms of disease. Future studies will require interrogation of a larger cohort to understand if these findings can be replicated, for NfL to be used as a biomarker on an individualized level to characterize the therapeutic window, and be applied to monitor presymptomatic infants and children not immediately initiating treatment to detect and treat disease activity during the prodromal phase.

Overall, the insights gleaned in this study gives weight to the hypothesis that the motor unit pool exists in 4 states and that there are relative degrees of salvageable and irreversibly damaged motor axons. For children with chronic SMA, there is evidence of a chronic axonopathy, as reflected by low CMAP secondary to a relatively higher proportion of irreversibly damaged motor neurons, and as reflected by relatively low sNfL values. In stark contrast, for newborns and those with 2 *SMN2* copies, high sNfL levels may reflect a component or “penumbra” of reversible but dysfunctional motor neurons, whereas high and stable CMAP levels align with the extent of the available motor unit pool. Targeting this state with SMN repletion is imperative to maximize the capacity of the motor unit pool.

Our results demonstrate a decline/stabilization in sNfL levels in individuals treated with nusinersen or risdiplam, whereas they have been observed to increase in individuals treated with onasemnogene abeparvovec.^5^ The transient increase linked to onasemnogene abeparvovec has been hypothesized to be secondary to central nervous system (CNS) inflammatory response against the adeno‐associated virus serotype 9 vector. Although the small numbers in nusinersen (monotherapy) treated subgroup preclude our ability to adjust for clinical variables, overall, the magnitude of reduction in sNfL with treatment may predict future motor gains, as seen in prior studies.^11^ The magnitude of sNfL reduction may reflect the extent of the motor unit pool available to be salvaged with SMN repletion, again aligning with the concept of a penumbra of motor axons on the cusp of neurodegeneration, which may be rescued by timely intervention.

The strength of this study is that it reflects a real‐world heterogeneous population which is representative of cohorts internationally.^36^, ^37^ Here, the set of baseline characteristics, combining sNfL and neurophysiological with clinical measures in a regression model provided a stronger assessment of ongoing disease biology and begins to conceptualize a penumbra. In this study, a subset of the cohort had CSF derived NfL values. As the field heads toward using sNfL as part of standards of care, the strong correlation between serum and CSF NfL values, as shown in this and prior studies, means that it is important to acknowledge that sNfL values can be interchangeably used with CSF.

Limitations of the study include the small numbers within the cohort, which is a common challenge within rare disease research. This is compounded by the clinical heterogeneity of the population which may have underpowered subgroup analyses. For example, whereas the size of effect of NfL suppression for individuals treated with nusinersen was large, it is likely that the small size for this subgroup resulted in low power for this effect. Thus, future studies to understand the aggregated role of these biomarkers in depicting the stages of disease and pharmacodynamic response are important. This may also include measurement of the motor unit pool through motor unit number estimation methodologies to elucidate the true balance between collateral reinnervation and denervation in disease and treatment states in SMA.^38^

Although reference values for healthy controls are available, the normative data used here for comparison purposes should be considered with caution, due to challenges with normalization between assays and the imperative for further work to obtain sNfL ranges specifically for a newborn population. In addition, investigations focused on other factors that impact sNfL levels in the early postnatal period should be undertaken. Future work should focus on collaborative efforts across national and international reference centers to increase the power of these findings.

## Author Contributions

Conception and design of the study, A.D, M.F, D.K; Acquisition and analysis of the data, A.D, K.H, L.B, JM.H, T.K, H.S, HL.T, E.T, N.B, N.N, M.K, M.F, D.K; Drafting the text and preparing the figures, A.D, M.F, D.K, N.B, N.N, JM.H. All authors have read and agreed to the published version of the manuscript.

## Potential Conflicts of Interest

A. D'Silva, L. Balaji, and M. Kiernan have received speaker's fees from Biogen. K. Herbert, J. He, T. Kandula, H. Sampaio, H. L. Teoh, E. Tantsis, N. Briggs, and N. Ning report no disclosures relevant to the manuscript. J. Sohn was a paid employee of Biogen at the time of the data analysis. D. Kariyawasam has received honoraria for advisory board participation from Biogen and speaker's fees from Biogen, Novartis, and Roche. M.A. Farrar is a site principal investigator for Roche and Novartis Gene Therapies Inc. clinical trials, and their institution has received funds for contract research related to the conduct of these trials. M.A. Farrar has received honoraria for advisory board participation from Biogen, Novartis, and Roche; has received speaker's fees from Biogen, Roche, and Novartis; and serves as a medical director on the board of Muscular Dystrophy NSW (not‐for‐profit). Although the immunoassays were carried out by Biogen (sponsors of nusinersen), all analysis and data interpretation were conducted independently of this company.

## Supporting information


**Supplementary Data S1.** Supporting Information.

## Data Availability

Individual de‐identified participant data will be made available post publication upon request and a signed data access agreement.
