# Supplementary material for: The Dynamics of Neurofilament Light Chain in Spinal Muscular Atrophy
Source: Ann Neurol. 2026 Mar 17;100(1):109–22. doi: 10.1002/ana.78207 (PMC13327556; doi:10.1002/ana.78207)
Supplement: Supplementary file 1 — Supplementary Data S1. Supporting Information. [file ANA-100-109-s001.docx]

**Supplementary Figure 1. Number and timing of longitudinal neurofilament light chain assessments in individuals undergoing nusinersen treatment.**

Biospecimen collection included 1–2 ml of serum from venepuncture. For children initiating intrathecal nusinersen a 5 ml aliquot of cerebrospinal fluid (CSF) was collected as part of standard clinical administration practice^31^. From the CSF samples, multiple aliquots (200–1000 µl) were stored frozen at – 80 °C. Blood was drawn in serum-separating tubes (SST) and serum extracted from blood clotted for 20 minutes by centrifugation at 1000 – 2000 x *g* for 10 minutes at 4 °C. Serum aliquots (200–500 µl) were stored frozen at – 80 °C.

For individuals receiving nusinersen treatment, biospecimens were collected at day 0, defined as prior to treatment, within the period of loading phase of the drug regimen (defined as day 14–63) and the maintenance phase of the dosing regimen (where 4 monthly intrathecal injections were administered).

**Supplementary methods**

*Neurofilament light chain analysis*

NfL concentrations in CSF and serum were measured on the Quanterix Simoa HD-X analyzer using the Simoa® NF-light immunoassay advantage kit V2 (Product 104073, LOT number 504327 and 503808) (Quanterix, Lexington, MA, USA) according to the manufacturer’s instructions (Supplementary methods). Broadly, the assay used two NfL specific monoclonal antibodies. Sample, paramagnetic capture beads coated with anti-NfL antibody, and a biotinylated detector antibody were combined. NfL molecules present in the sample were captured by the anti-NfL antibody coated paramagnetic capture beads and bound with the biotinylated antibody detector simultaneously.

Following a washing step, a conjugate of streptavidin-beta-galactosidase (SBG) was mixed with the capture beads. The captured NfL became enzymatically labelled when the SBG bound to the biotinylated detector antibodies. A second wash was performed, and the capture beads were resuspended in a resorufin beta-D-galactopyranoside (RGP) substrate solution and transferred to the Simoa Disc. Individual beads were then sealed within microwells in the array. Once NfL was captured and labelled on the bead, ß-galactosidase hydrolyzed the RGP substrate in the microwell into a fluorescent product that provided the signal for measurement. This signal was detected and counted by the Simoa optical system. The concentration of NfL was interpolated from a standard curve. (Package insert: Simoa NF-Light Advantage Kit. Quanterix Corporation; 2019).

Prior to sample testing, three rounds of Endogenous Quality Control (EQC) pre-screening were carried out, which was followed by kit lot qualification (i.e., testing of 6 kits for reproducibility of five levels of QCs (3x buffered QCs and 2X EQCs) and calibrants). Once qualification was complete, the data was used to establish nominal concentration for five levels of QCs that were subsequently included in sample testing to monitor assay performance.

**Supplementary Table 1. Demographics and clinical characteristics of individuals with SMA who did not initiate immediate treatment after diagnosis.**

| ***Clinical Characteristics*** | ***Study population (n = 7)*** |
| --- | --- |
| Sex (n, %) |  |
| Male | 2 (28.6) |
| Female | 5 (71.4) |
| Modality of diagnosis (n, %) |  |
| Newborn screening | 5 (71.4) |
| Cascade testing (family history) | 2 (28.6) |
| *SMN2*copy number (n, %) |  |
| 3 | 4 (57.1) |
| 4 | 3 (42.9) |
| Age of diagnosis, median, (range) days | 17 (7 – 15,412) |
| Age at last follow up, median, (range) days | 1725 (372 – 16,872) |

**Supplementary Table 2. Clinical monitoring of presymptomatic individuals with ≥3 *SMN2* for whom treatment was not initiated immediately.**

|  | | **First assessment** | | **Follow up assessment** | | **Latest assessment** | |
| --- | --- | --- | --- | --- | --- | --- | --- |
| **Participant number, diagnostic modality** | ***SMN2*copies** | **Age at assessment** | **Clinical and neurophysiological assessments** | **Age at assessment** | **Clinical and neurophysiological assessments** | **Age at assessment** | **Clinical and neurophysiological assessments** |
| 1  NBS | 3 | 11 days | CHOP-INTEND = 60 | 7 weeks | CHOP-INTEND = 62 | 10 weeks | CHOP-INTEND = 63 |
|  |  |  | HINE-2 = 2 |  | BSID3 = 12 |  | BSID3 = 16 |
|  |  |  | ulnar CMAP =  3.6 mV |  | HINE-2 = 2 |  | HINE-2 = 5 |
|  |  |  | EMG: MUPS normal |  | ulnar CMAP =  43 mV |  | ulnar CMAP =  5.6 mV |
|  |  |  | NA |  | EMG: MUPs normal |  | EMG: high amplitude MUPs, up to 1400 µV |
| 2  NBS | 3 | 7 weeks | CHOP-INTEND = 64 | 18 weeks | CHOP-INTEND = 64 | 35 weeks | CHOP-INTEND = 64 |
|  |  |  | HINE-2 = 5 |  | BSID3 = 8 |  | BSID3 = 9 |
|  |  |  | ulnar CMAP =  6.9 mV |  | HINE-2 = 13 |  | HINE-2 = 16 |
|  |  |  | EMG: MUPS normal |  | ulnar CMAP =  7.1 mV |  | ulnar CMAP =  9.3 mV |
|  |  |  | NA |  | EMG: MUPS normal |  | EMG: MUPs normal |
| 3  NBS | 3 | 9 weeks | CHOP-INTEND = 64 | 34 weeks | CHOP-INTEND = 64 | 62 weeks | CHOP-INTEND = 64 |
|  |  |  | HINE-2 = 2 |  | BSID3 = 7 |  | HINE-2 = 22 |
|  |  |  | ulnar CMAP =  4.3 mV |  | HINE-2 = 14 |  | ulnar CMAP =  6.8 mV |
|  |  |  | EMG: MUPS normal |  | ulnar CMAP = 5  .5 mV |  | NA |
| 4  Cascade testing | 3 | 42 months | HINE-2 = 26 | 44 months | HINE-2 = 26 | NA | NA |
|  |  |  | HFMSE = 64 |  | HFMSE = 64 |  |  |
|  |  |  | EMG: MUPS normal |  | NA |  |  |
| 5  NBS | 4 | 10 days | HINE-2 = 2 | 10 months | HINE-2 = 18 | 4.5 years | HINE-2 = 26 |
|  |  |  | ulnar CMAP =  4.7 mV |  | NA |  |  |
| 6  NBS | 4 | 8 days | CHOP-INTEND = 56 | 15 weeks | CHOP-INTEND = 64 | 30 weeks | CHOP-INTEND = 64 |
|  |  |  | ulnar CMAP =  4.3 mV |  | BSID3 = 12 |  | BSID3 = 15 |
|  |  |  | EMG:  MUPS normal |  | ulnar CMAP =  9.3 mV |  | HINE-2 = 20 |
|  |  |  | NA |  | EMG: MUPS normal |  | ulnar CMAP =  7.8 mV |
|  |  |  | NA |  | NA |  | EMG: Excess of large broad motor units |
| 7  Cascade testing | 4 | 42 years | HFMSE = 66 | 46 years | HFMSE = 66 | NA | NA |
|  |  |  | NA |  | ulnar CMAP =  APB 5.4 mV |  |  |
|  |  |  | NA |  | EMG: MUPS normal |  |  |

Monitoring was based on the recommendations of the United States SMA expert working group, including CHOP-INTEND, HINE-2, BSID3, CMAP and EMG based on age and tolerability, compliance and clinical resources at each assessment. All assessments undertaken at each visit are reported. Those not reported were due to poor compliance, resources not available or parental refusal.  BSID III: Bayley Scales of Infant and Toddler Development 3^rd^ edition, CHOP INTEND: Children’s Hospital of Philadelphia Infant Test of Neuromuscular Disorders, CMAP: Compound muscle action potential, EMG: Electromyography, HFMSE: Hammersmith Functional Motor Scale – Expanded, HINE: Hammersmith Infant Neurological Examination, MUPs: motor units potentials; NA: Not applicable; NBS: newborn screening

Follow up was undertaken for the 5 individuals that initiated treatment, ranging from 4.5 to 55.4 months post treatment initiation. Two infants (participant 1 and 6) had neurogenic changes on EMG, enabling the initiation of treatment, even though motor function measures and development remained age appropriate. For these infants, follow-up of 2 years and 3 months post treatment respectively, demonstrated motor function outcomes consistent with expectations for typically developing children. Similarly, for the 3 individuals that started treatment whilst presymptomatic, motor function remained within normal trajectories despite evidence of subclinical disease activity as denoted by elevated pretreatment serum neurofilament light chain (sNfL) levels. Treatment has not been initiated in two individuals; one has been lost to follow up aged 5.5 years, and the other is presymptomatic aged 46 years. The individual case summaries are detailed below.

**Participant 1:**

This was a term, female infant, identified through NBS. Diagnostic testing confirmed SMA and 3 *SMN2* copies. Initial assessments at 2 and 7 weeks demonstrated no symptoms, a normal motor examination, increasing motor function and CMAP amplitudes. At 10 weeks, subtle symptoms and signs on examination were consistent with SMA but not definitive - namely, upper limb tremor when crying and regression in chin tuck on pull to sit manoeuvre: clinical observation also noted decreased and reduced quality of lower limb movements. However, motor function assessments and CMAP amplitudes continued to increase. EMG at 10 weeks demonstrated neurogenic changes with high amplitude, broad motor unit potentials. Concurrently sNfL levels increased approximately 17-fold from initial values. Following initiation of therapy at 10 weeks, there was a 29-fold decline of sNfL to 18 pg/mL at age 16 weeks and further decrease to 5.1 pg/mL by age 24 weeks. At follow up, she demonstrated the acquisition of motor milestones within timeframes for typically developing children, sitting independently at 7 months and walking independently at 14 months, with increasing motor function scores. At 2.25 years, she had a normal clinical examination, she achieved a BSID-3 score of 52 (scaled score 9, 1 standard deviation (SD) below the mean).

**Participant 2:**

This healthy term male newborn was identified with SMA through NBS. Diagnostic testing confirmed SMA with 3 *SMN2*copies. The infant continued to have a normal clinical examination, with increases in motor function scores and acquisition of motor milestones and normal neurophysiology at 8 months. At this time changing reimbursement criteria led to treatment whilst presymptomatic with onasemnogene abeparvovec. At the time of treatment initiation sNfL was 11-fold higher than the 95^th^ centile for neurologically healthy age-matched peers. During follow up he commenced walking independently at 11 months but continued to intermittently toe walk (family history of same). At last assessment aged 2.4 years he had a normal clinical examination HFMSE score was 62/66 and gross motor skills on the BSID-3 were 2 SD above the mean.

**Participant 3:**

This female infant was identified with SMA through NBS. Diagnostic testing confirmed SMA and 3 *SMN2* copies. There was a maternal family history of progressive weakness requiring wheelchairs for mobility, without specific diagnosis. During infancy there were no reported symptoms, normal motor examination and increasing CMAP amplitudes. EMG assessment was declined by parents. She started walking independently aged 14 months. Changing access to disease modifying therapies led to treatment with onasemnogene abeparvovec aged 15 months whilst presymptomatic however pretreatment sNfL levels were 14-fold higher than the 95^th^ centile for neurologically healthy age matched peers at 14 months, an increase from the previous measurement 12 months earlier. At 5.5 years she had no symptoms, a normal clinical examination and HFMSE score was 64.

**Participant 4:**

This female had diagnostic testing for SMA at age 3.5 years, following the diagnosis of her symptomatic older brother. This showed homozygous deletions of *SMN1*exon 7 and 3 *SMN2* copies. She had no symptoms, a normal motor examination and HFMSE was 64/66. Pretreatment neurofilament light chain levels increased from the 75-90^th^ centile at 3.5 years to the 90-95^th^ centiles at 3.7 years for neurologically healthy age matched peers. She initiated treatment whilst presymptomatic at age 3.7 years. At age 7.3 years she had no symptoms, a normal clinical examination and HFMSE score was 66/66.

**Participant 5:**

This female infant was identified with SMA through NBS. Diagnostic testing confirmed SMA and 4 *SMN2* copies. Regular clinical assessments, including neurological examination and motor function assessments were undertaken every 3-6 months until age 2 years and were age appropriate. She walked independently at 12 months. The parents declined neurophysiological studies after the initial assessment. Assessments after age 2 years were offered to the family every 6-12 months and occurred at ages 3 and 4.5 years. These showed normal motor examination and function. sNfL levels at 10 months and 4.5 years were 1-2 fold higher than the 95^th^ centile for age-matched neurologically health children. The parents declined further follow up at age 5.5 years, reporting that the child was healthy and age appropriate.

**Participant 6:**

This male infant, born at 40 weeks was identified through NBS. Diagnostic testing confirmed SMA with 4 *SMN2* copies. He was febrile in the newborn period and treated with intravenous antibiotics. No infection was identified on full septic work up; however, the family was unwell with gastroenteritis. Clinical examination was normal; he sat independently at 6 months; motor function scores increased during follow up. At 9 months, neurophysiology demonstrated a decrease in CMAP amplitude compared to previous assessment (however within normal range for age) and neurogenic changes. At this time serum sNfL was 1-2 fold higher than the 95^th^ centile for age-matched neurologically healthy infants. Risdiplam was commenced, after which, sNfL levels reduced from 19 pg/ml to 12 pg/mL at age 11 months (95^th^ centile for healthy age-matched infants =18.2 pg/ml). At 10 months he was stood and walked independently and continued to be presymptomatic 3 months post treatment.

**Participant 7:**

Following the diagnosis of their newborn with SMA (Participant #3), the parents underwent *SMN1* testing for reproductive planning. This 42-year-old mother was diagnosed with SMA (biallelic exon 7 *SMN1* deletions) and had 4 *SMN2* copies. The positive modifier in *SMN2* c.859G>C was not identified on sequencing. She had no symptoms, HFMSE was 66/66, CMAP amplitude and sNfL levels were within normative reference ranges for age throughout the 4-year follow up period.
